# Effects of pre-exercise ingestion of a carbohydrate-electrolyte gel on cycling performance

**DOI:** 10.1186/1550-2783-8-S1-P28

**Published:** 2011-11-07

**Authors:** Mark ET Willams, Chris DJ Toy, Andrew M Cox

**Affiliations:** 1 Department of Sport and Exercise Sciences. University of Chichester, United Kingdom

## Background

Exercise performance can benefit from pre-exercise ingestion of carbohydrate-electrolyte drinks. Carbohydrate-electrolyte gels may provide a convenient and effective energy source for subsequent exercise bouts, but supportive evidence needs to be provided. We examined the effect of pre-exercise ingestion of a commercial carbohydrate-electrolyte gel on cycling performance.

## Methods

Following an overnight fast, healthy males (n = 12, age: 24 ± 7 yr, height: 181 ± 6 cm, body mass: 78.1 ± 9.4 kg, VO_2max_: 47.6 ± 7.1 mL·kg^-1^·min^-1^, W_max_: 316 ± 51 W) cycled steady state (40 min, SS1, 56 ± 4%W_max_, SRM Ergometer) followed by a time trial (15 min, TT1,Wattbike cycle ergometer), a 2 hour passive recovery, and cycled steady state (20 min, SS2, power equal to SS1) followed by a time trial (15 min, TT2). Participants ingested either placebo (P, low-caloric gel, equal in flavour) or Maxifuel’s Viper® Active Gel (V, 65 gram equal to one gel) (Maxinutrition Ltd, Hemel Hempstead, UK), 15 min pre-SS1 (+250 ml water), 0 hr post-TT1 (+750 ml water), 1 hr post-TT1 (+250 ml water), and 15 min pre-SS2 (+250 ml water). Maxifuel’s Viper® Active Gel contains 22 g maltodextrin, 11.2 g sucrose, 1.5 g dextrose, 0.8 g fructose and 0.1g sodium per 100g). Experimental design was double-blind and randomized. Carbohydrate oxidation was calculated with stoichiometric equations from Jeukendrup & Wallis. Two-way ANOVA with post-hoc t-tests were used for analysis with significance accepted at p < 0.05.

## Results

During SS1, heart rate, oxygen uptake, respiratory exchange ratio, rating of perceived exertion, plasma lactate and carbohydrate oxidation were not different between conditions. There was a trend for blood glucose (mmol·L^-1^) with Viper during SS1 to be higher at 0 min (P: 4.26 ± 0.21, V: 6.36 ± 0.76) and 10 min (P: 3.89 ± 0.37, V: 4.98 ± 0.70), and lower at 20 min (P: 3.89 ± 0.47, V: 3.12 ± 0.69) and 30 min (P: 3.92 ± 0.45, V: 3.12 ± 0.69). During SS2, heart rate, oxygen uptake, rating of perceived exertion and plasma lactate were not different between conditions. Blood glucose (in mmol·L^-1^) with Viper during SS2 was higher at 0 min (P: 3.80 ± 0.40, V: 5.33 ± 0.77) and 10 min (P: 3.56 ± 0.40, V: 4.10 ± 0.55). Respiratory exchange ratio was higher during SS2 for Viper at 5 min (P: 0.90 ± 0.09, V: 0.99 ± 0.08). Carbohydrate oxidation (g·min^-1^) during SS2 was higher with Viper at 5 min (P: 2.11 ± 0.84, V: 2.97 ± 0.71). Cycling distance during TT1 and TT2 was 3.1% (P: 9467 ± 963 m, V: 9741 ± 817 m) and 3.4% (P: 9375 ± 943 m, V: 9667 ± 746 m) higher with the carbohydrate-electrolyte gel ingestion.

## Conclusion

It is concluded that pre-exercise ingestion of a 65 gram commercial carbohydrate-electrolyte gel with multiple carbohydrates benefits cycling performance. In addition, the ingestion of the carbohydrate-electrolyte gel during recovery enhanced subsequent cycling performance. The consumption of commercial carbohydrate-electrolyte gels with different carbohydrates may be beneficial for athletes with multiple daily training sessions.

## References

[B1] Jeukendrup Wallis 2005

